# Characterization of Hepatitis B Virus Integrations Identified in Hepatocellular Carcinoma Genomes

**DOI:** 10.3390/v13020245

**Published:** 2021-02-04

**Authors:** Pranav P. Mathkar, Xun Chen, Arvis Sulovari, Dawei Li

**Affiliations:** 1 Department of Microbiology and Molecular Genetics, University of Vermont, Burlington, VT 05405, USA; Pranav.Mathkar@uvm.edu (P.P.M.); arvissulovari@gmail.com (A.S.); 2 Institute for the Advanced Study of Human Biology, Kyoto University, Kyoto 606-8501, Japan; 3 Cajal Neuroscience Inc., Seattle, WA 98102, USA; 4 Department of Biomedical Science, Charles E. Schmidt College of Medicine, Florida Atlantic University, Boca Raton, FL 33431, USA

**Keywords:** viral integration, virome-wide detection, VIcaller, integration allele fraction, hepatocellular carcinoma (HCC), hepatitis B virus (HBV)

## Abstract

Hepatocellular carcinoma (HCC) is a leading cause of cancer-related mortality. Almost half of HCC cases are associated with hepatitis B virus (HBV) infections, which often lead to HBV sequence integrations in the human genome. Accurate identification of HBV integration sites at a single nucleotide resolution is critical for developing a better understanding of the cancer genome landscape and of the disease itself. Here, we performed further analyses and characterization of HBV integrations identified by our recently reported VIcaller platform in recurrent or known HCC genes (such as *TERT*, *MLL4*, and *CCNE1*) as well as non-recurrent cancer-related genes (such as *CSMD2*, *NKD2*, and *RHOU*). Our pathway enrichment analysis revealed multiple pathways involving the alcohol dehydrogenase 4 gene, such as the metabolism pathways of retinol, tyrosine, and fatty acid. Further analysis of the HBV integration sites revealed distinct patterns involving the integration upper breakpoints, integrated genome lengths, and integration allele fractions between tumor and normal tissues. Our analysis also implies that the VIcaller method has diagnostic potential through discovering novel clonal integrations in cancer-related genes. In conclusion, although VIcaller is a hypothesis free virome-wide approach, it can still be applied to accurately identify genome-wide integration events of a specific candidate virus and their integration allele fractions.

## 1. Introduction

Hepatocellular carcinoma (HCC), a primary liver malignancy and leading cause of cancer-related deaths, is a major global health concern. The onset of HCC is often preceded by chronic liver conditions (such as hepatitis, cirrhosis or fibrosis [1,2]), which can further complicate anti-cancer treatment regimens [3]. Extensive research has established that chronic Hepatitis B virus (HBV) infection is a leading risk factor for the initiation and progression of HCC [4] and may account for approximately half of all HCC cases [5]. With about 400 million global cases of HBV infection [5], HBV-associated HCC remains highly prevalent, particularly in parts of Asia and Sub-Saharan Africa [6]. Despite the strong correlation between HBV infections and HCC onset, the precise genetic and genomic mechanisms underlying this relationship are still poorly understood [5].

Following infection, HBVs commonly integrate their DNA into the human genome. Such integration events have been identified in 75–90% of HCC tissues [7]. These integrations may further lead to development of host-virus fusion transcripts, particularly if the integrations occur within genic regions. The HBV genome is circular and partially double stranded and is approximately 3200 base pairs (bp) in length. It contains four overlapping open reading frames, namely surface, core, polymerase, and X [8]. The HBV X region, located near the nucleotide 1800 (nt 1374–1838) region on the HBV reference genome [9], is often retained following integration events and is selectively over-expressed in HCC [10,11]; thus, it may contribute to the high rate of metastasis in HBV-associated HCC patients [12]. Systematic research on the identification and characterization of the patterns of integration breakpoints on both the human and HBV genomes is critical for understanding the mechanistic processes of HBV integrations. We recently found that the cellular proportion of each integration event, which can be estimated based on its integration allele fraction, serves as important evidence to identify integration events involved in early-stage tumorigenesis [13]. Such analyses may provide new opportunities to understand the genomic landscape of HBV integrations and their involvement in the early stages of HCC development, which may help identify new diagnostic biomarkers and potential targets for therapeutic intervention.

An increasing number of studies have focused on the detection of infectious pathogens using cancer high-throughput sequencing (HTS) data [14]. HTS reads uniquely mapped to a viral reference genome are strong evidence of the presence of the virus in a sample; however, this analysis is compounded by two major practical challenges. First, the detected virus-mappable reads may have been derived from environmental microbes, synthetic DNA [15], or commonly-used cell lines such as HeLa cells [16]. Second, the presence of virus-mappable reads alone may not be conclusive for its role in tumorigenesis since viral infection may have occurred after oncogenesis [16]. To overcome these challenges, we recently developed a novel strategy and bioinformatics platform, viral integration caller (VIcaller), for identifying clonal viral integrations in the human genome and providing estimated integration allele frequencies [13]. We recently reported our preliminary findings on the analysis of whole genome sequencing (WGS) data from 88 HBV-associated HCC patients [9] using the VIcaller approach, and demonstrated high sensitivity and precision for the identification of integration events [13]. In this study, we report our in-depth analyses of these HBV integration events detected by VIcaller, as well as the characterization and pathway enrichment analyses of these integration events.

## 2. Methods

### 2.1. Detection of HBV Integrations Using VIcaller

A total of 88 HCC samples with both tumor and paired normal tissues were previously analyzed using the VIcaller platform [13]. In brief, we first submitted the paired-end reads in FASTQ format to VIcaller’s “detect” function to screen for integrations of the candidate virus, i.e., HBV (NC_003977.2), with the parameters: “-d WGS -m standard -r -a -q 20”. We then validated all identified candidate HBV integrations using the VIcaller “validate” function with the default parameters. Only the successfully validated HBV integration candidates were included for further analyses. Lastly, we calculated the integration allele fraction of each detected HBV integration event using the VIcaller “calculate” function with default parameters. To identify novel HBV integration events in the 88 HCC samples, we subsequently compared our detected HBV integrations with those presented by Sung et al. [9]. The resulting HBV integrations were then classified as “consistent”, if detected both by VIcaller and Sung et al. “novel”, if detected by VIcaller but not by Sung et al., and “missing” if detected by Sung et al. but not VIcaller [13].

### 2.2. Repeat Sequence Comparative Analysis

The repeat sequence annotation file generated by RepeatMasker [17] was downloaded from the UCSC Genome Browser (build hg19). The repeat density was examined using the BEDtools2 window function [18], and we determined the proportion of repeat sequences covering a 2000 bp window centered at each HBV integration site. We compared the repeat densities of novel, missing, and consistent HBV integrations with random positions across the human genome using a student’s *t*-test.

### 2.3. Upper and Lower Breakpoint Analyses

The genomic coordinates of the upper and lower breakpoints of each integration on the HBV reference genome (NC_003977.2) were analyzed separately. The HBV upper breakpoint is the genomic coordinate of the sequence junction connected upstream (5′) orientation on the human sequence, while the lower breakpoint is the other HBV sequence junction connected downstream (3′) on the human sequence. When the breakpoint locations were detected from chimeric reads only (without split reads; chimeric and split reads were depicted in Supplementary Figure S1 of our previous paper [19]), the nearest location to the breakpoint was adopted. HBV integrations with one breakpoint and those with two breakpoints were analyzed separately. The lengths of inserted HBV genomes or sequences were estimated based on the HBV integration events with both upper and lower breakpoints detected.

### 2.4. Gene Ontology and Pathway Enrichment Analyses of Novel HBV Integrations

We first converted the identified novel HBV integrations into variant call format, treating HBV integrations as insertions relative to the human reference genome, and then performed functional annotation using CADD [20] (https://cadd.gs.washington.edu). For the human genes in which the integrations occurred, we manually obtained and curated protein functions and associated diseases or biological functions through literature searches. To identify novel pathways involving genes other than the known HCC-associated genes (i.e., *TERT*, *MLL4*, and *CCNE1*), our pathway enrichment analysis was carried out based only on non-recurrent human genes with HBV integrations (i.e., genes observed only once in our examined samples) using the KEGG pathway database [21,22], as described in our previous paper [22]. Briefly, the overrepresentation of pathway-associated genes from our gene set was modelled according to a hypergeometric distribution function to generate enrichment *p* values; and the enrichment ratio was defined as expected divided by observed number of genes per pathway.

### 2.5. Integration Allele Fraction Analysis

The allele fraction of each HBV integration detected in the tumor and paired normal tissues of each subject (i.e., HCC sample) was calculated by VIcaller. The HCC samples were ranked first by the highest allele fraction in the tumor tissues; then by the highest in the normal tissues having no HBV integrations detected in the tumors. The HBV integrations with high allele fractions (i.e., ≥90%), but which were supported by only a very small number of reads (i.e., fewer than four supporting reads), were excluded from subsequent analyses.

### 2.6. Tumor Grade Analysis

The tumor pathology grades (i.e., poor, moderate, and high levels) were obtained for each subject from the Sung et al., study [9]. A student’s *t*-test was used to compare the average numbers of HBV integrations between each pair of the distinct pathology grade groups. All 88 analyzed HCC patients were from chronically HBV infected patients. Additional demographic and clinicopathologic characteristics of the samples were described in Supplementary Table S1 of the Sung et al., study [9].

## 3. Results

### 3.1. Analyses of Novel HBV Integrations Identified by VIcaller

**Figure 1:**
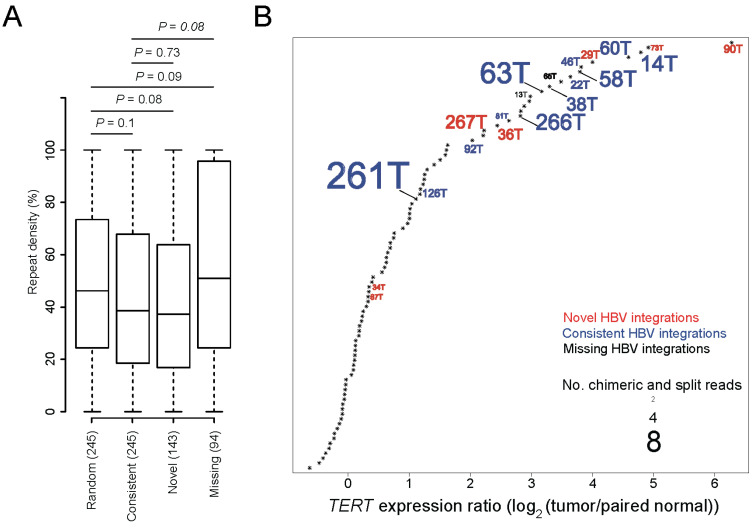
Accuracy of the novel hepatitis B virus (HBV) integrations detected by VIcaller. (**A**) Comparison of the repeat sequence density values among the novel, consistent, and missing HBV integration events and randomly selected genomic positions. “Novel” HBV integrations are those detected only by VIcaller but not Sung et al. “Consistent” HBV integrations are those detected by both VIcaller and Sung et al. “Missing” HBV integrations are those detected only by Sung et al. but not VIcaller. The *p* values were computed using a student’s *t*-test. (**B**) Most of the *TERT* gene expression levels of the novel HBV integrations are significantly up-regulated and thus ranked among the top upregulations. Only samples with HBV integrations in *TERT* are labeled in the plot. Red, blue, and black correspond to novel, consistent, and missing integrations, respectively. The font size has been scaled to reflect the number of supporting reads per integration.

In general, HBV integrations identified in genomic repetitive regions are considered less accurate than those identified in other genomic regions due to higher alignment errors of HTS reads in these regions (Supplementary Figure S1). We combined the HBV integrations identified from HBsAg-positive and negative tumor and normal tissues, and then compared the density values of repeat sequences among four groups, including our identified novel and consistent HBV integrations, missing integrations, and randomly sampled genomic positions. We found that the novel and consistent HBV integrations showed decreased repeat sequence density compared to the randomly sampled positions, although the *p* values were statistically insignificant (*p* values = 0.08 and 0.1, respectively). By comparison, the repeat sequence density of the missing integrations trended higher than our randomly sampled genomic positions and consistent HBV integrations (*p* values = 0.09 and 0.08, respectively) (Figure 1A). We further observed that most of the samples with novel HBV integrations (e.g., samples 90T and 73T) showed significantly upregulated *TERT* gene expression levels, implying that the novel HBV integrations detected by VIcaller are accurate and *TERT* upregulation is likely involved in the disease mechanism in these samples (Figure 1B).

### 3.2. Charaterization of HBV Integration Breakpoints

We first characterized the upper and lower breakpoints on the HBV reference genome of the 388 HBV integrations [13] identified in the tumor and paired normal tissues of the 88 subjects. We found that both upper and lower breakpoints were highly enriched at ~1.8 kb, with the upper breakpoints also enriched between 1.8 kb and 3.2 kb compared to the lower breakpoints (Supplementary Figure S3A). These patterns were consistent between the HBV integrations with one breakpoint, and those with two identified breakpoints (Supplementary Figure S3B,C).

**Figure 2:**
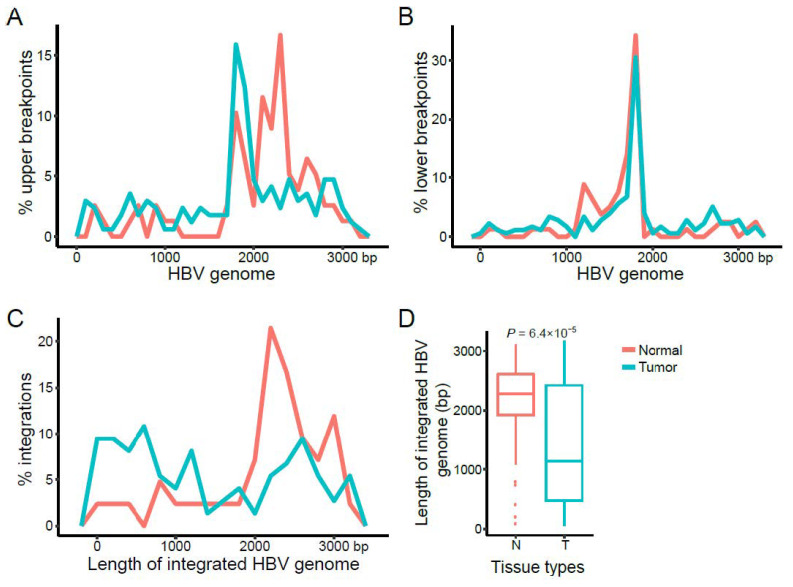
Characteristics of the HBV breakpoints and inserted HBV genome length. Distribution of the (**A**) upper and (**B**) lower breakpoints on the HBV genome. (**C**,**D**) Distribution and comparison of the lengths of the inserted HBV sequences between tumor and normal tissues. The *p* values were computed using a student’s *t*-test.

We then characterized breakpoint hotspots of HBV integrations between the tumor and paired normal tissues. The upper and lower breakpoints on the HBV genome were analyzed separately. We observed distinct patterns for the upper breakpoints on the HBV genome between the tumor (at ~1.8 kb) and normal (at ~2.2 kb, core gene) tissues (Figure 2A). In contrast, the lower breakpoint hotspots were located at ~1.8 kb (core and X genes) in both tumor and normal tissues (Figure 2B), which was consistent with previous results [9]. Moreover, we found that the length of inserted HBV sequences detected in tumor tissues were significantly shorter than those detected in normal tissues (*p* value = 6.4 × 10^−5^; Figure 2C,D); and the HBV integrations in the normal tissues were prone to have more complete HBV genome sequences, with an enrichment of the upper breakpoints at ~2.2 kb.

### 3.3. Annotation of Novel Non-Recurrent Genic HBV Integrations and Gene Pathway Analysis

We performed gene annotation analysis of all novel non-recurrent HBV integrations, i.e., integrations in genes other than *TERT*, *MLL4*, and *CCNE1*. We identified 48 genic HBV integrations in 18 tumor samples and 29 normal samples (Table 1). For example, we detected an integration event in the intronic region of the *CSMD2* gene, supported by five chimeric and split reads. *CSMD2* may act as a tumor suppressor gene for colorectal cancer [23]. We detected another integration in the *RHOU* gene, which has been associated with basal cell carcinoma [24]. We also detected an integration in the intronic region of *NKD2* supported by four chimeric and split reads. *NKD2* is a wnt antagonist shown to escort TGF-α-containing exocytic vesicles [25]. Dysregulation of *NKD2* has been suggested to be involved in tumorigenesis of multiple cancers, including gastric cancer [26], esophageal cancer [27], and HCC [28], among others.

We then carried out gene pathway enrichment analysis for all the non-recurrent genes with any HBV integrations using the KEGG pathway database (Table 2). We found enrichment of multiple pathways involving the alcohol dehydrogenase 4 (*ADH4*) gene. Among these pathways, the retinol metabolism pathway was the most significant, followed by the tyrosine metabolism and fatty acid metabolism pathways (*p* = 0.0005, 0.004, and 0.004; and enrichment ratios = 43.95, 45.73, and 43.61, respectively). We also observed enrichment of pathways involving *ITGA11*, such as focal adhesion and extra cellular matrix-receptor interaction (*p* = 0.004 and 0.006; and enrichment ratios = 14.06 and 22.06, respectively). Additionally, other important pathways related to metabolism were also significantly enriched, such as arachidonic acid metabolism, drug metabolism, and purine metabolism (*p* = 0.004, 0.005, and 0.01; and enrichment ratios = 31.78, 25.69, and 11.57, respectively).

### 3.4. Characteristics of Integrations Involved in Early-Stage Tumorigenesis

**Figure 3:**
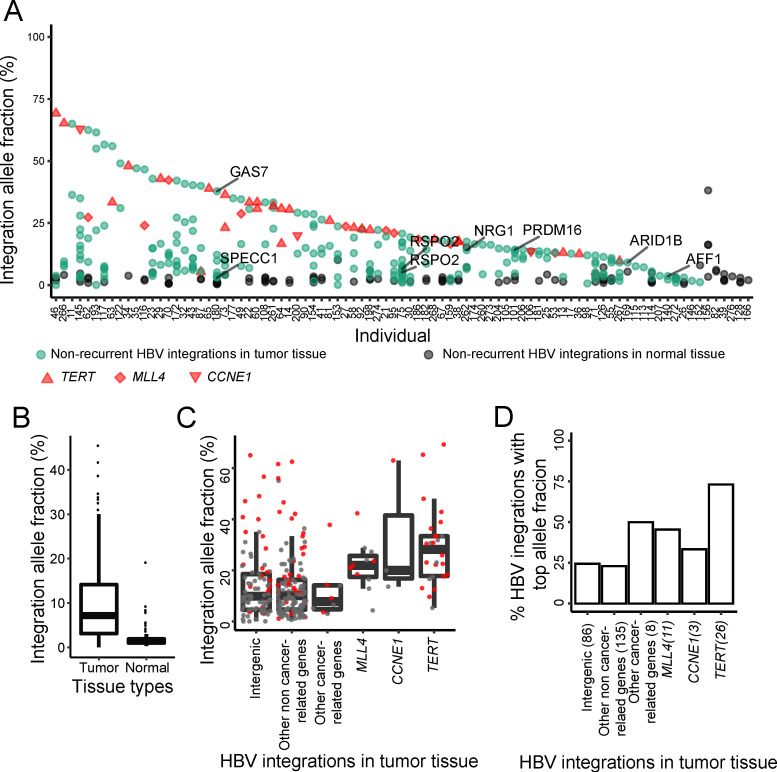
Integration allele fractions of HBV integrations in tumor and paired normal tissues. (**A**) HBV integrations in tumor and its paired normal tissue of each subject. The HBV integrations in the *TERT*, *MLL4*, and *CCNE1* (recurrent) genes are shown in red and the HBV integrations in other (non-recurrent) cancer-related genes are in green. (**B**) Comparison of the HBV integration allele fractions between tumor and normal tissues. (**C**) Comparison of the HBV integration allele fractions among different groups (the integration event with the top allele fraction was used for each subject). (**D**) Comparison between the number of HBV integrations with top cellular proportion between different groups.

We analyzed the integration allele fraction of each HBV integration event in both the tumor and its paired normal tissues for each sample. We found that HBV integrations had higher allele fractions in the tumor tissue compared to its paired adjacent normal tissue in nearly all samples (Figure 3A,B). We excluded six HBV integration events because each had fewer than four supporting reads, even they showed extremely high integration allele fractions (i.e., 100% for five integrations and ~90% for one integration). The HBV integrations in the three well-known recurrent genes, *TERT*, *MLL4*, and *CCNE1*, always had the highest integration allele fractions [13] in each sample. Besides the integrations in these three genes, we identified seven HBV integrations with high integration allele fractions in other genes, most of which have been reported to be associated with cancer, including *GAS7*, *SPECC1* (*NSP*), *RSPO2*, *NRG1*, *PRDM16*, *ARID1B*, and *AFF1*, as initially described in our recent method paper [13]. The HBV integrations in *GAS7*, *NRG1*, *PRDM16*, and *ARID1B* are among the highest integration allele fractions in each sample and no HBV integrations were found in any of the three recurrent genes (i.e., *TERT*, *MLL4*, and *CCNE1*) in these samples. In comparison to the three recurrent genes, the non-recurrent cancer-related genes had comparably high HBV integration allele fractions, e.g., also significantly higher than intergenic integrations and integrations in non-cancer-related genes (Figure 3C,D). The results imply that the HBV integrations in these cancer-related genes may also be involved in the early-stage tumorigenesis and potentially play vital roles in HCC.

### 3.5. Comparison of HBV Integration Abundance among Tumor Grades

**Figure 4:**
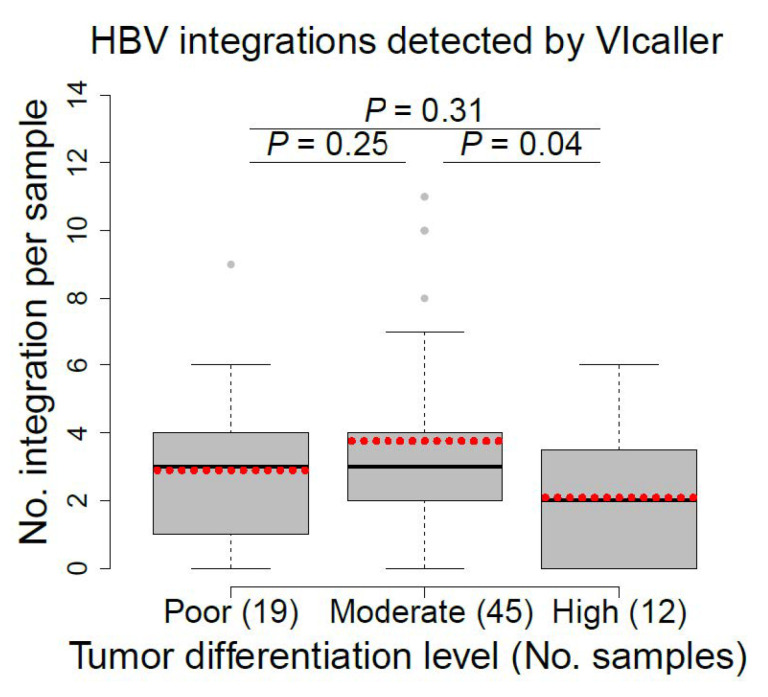
Comparison of the number of HBV integrations detected by VIcaller among poor, moderate, and highly differentiated tumors. Student’s *t* test was used to compare each group pair. The dotted red line indicates the average number of HBV integrations for each patient group.

Well-differentiated cancer cells tend to grow and spread more slowly than poorly differentiated or undifferentiated cancer cells [69]. We compared the number of HBV integrations per sample detected by VIcaller among three tumor groups, including poor, moderately, and highly differentiated tumors. The numbers of HBV integrations distinguished highly differentiated tumors (mean ± standard deviation = 2.1 ± 2.1) from moderately differentiated tumors (3.8 ± 3.5) with *p* value of 0.04 (Figure 4). This preliminary finding, if proven, may be crucial as patients with highly differentiated tumors have better prognosis than those with moderately differentiated tumors. By comparison, no statistically significant difference was reported based on the HBV integrations detected by Sung et al. [9] (*p* > 0.05; Supplementary Figure S4).

## 4. Discussion

Chronic HBV infections play a considerable role in the pathophysiology of HCC. The large-scale efforts of vaccination have reduced the incidence of HBV infections; however, chronic HBV infections are still persistent in 3.5% of the global population [70]. HBV infections often subsequently lead to HBV sequence integrations in the human genomes. It is imperative to accurately identify and characterize precise HBV integration events. Understanding the HBV integration sites in HCC genomes may provide new targets for therapeutic development. In this study, we carried out a comprehensive analysis of the HBV integrations that we previously detected in 88 HCC patients using our virome-wide approach, VIcaller. We compared our findings with those reported in a previous study that used an HBV-specific approach. We focused on the novel integration events identified by our VIcaller approach. Since the contributions of *TERT* and *MLL4* have been reported previously [7,71,72,73], we primarily examined and characterized the novel non-recurrent genic HBV integrations. Furthermore, it is notable that the VIcaller method has diagnostic potential through discovering novel integrations in known cancer-related genes. For example, we found intronic *TERT* integrations that have not been reported previously in samples 73T and 90T, and these findings provide evidence supporting a possible genetic diagnosis or explanation (which still needs further investigation) for the corresponding HCC patients for the first time.

We identified HBV integrations with high integration allele fractions in tumors in seven non-recurrent cancer-related genes, including *GAS7* [74,75], *NSP* [76], *RSPO2* [77,78], *NRG1* [79], *PRDM16* [80], *ARID1B* [81], and *AFF1* [82]. *GAS7* has been shown to be directly regulated by P53 and is part of a critical mechanism that mediates breast cancer metastasis [74]; *NRG1* fusions were identified as drivers for lung adenocarcinoma [79]; And the involvement of *PRDM16* in leukemia [80], *ARID1B* in ovarian cancer [81], and *AFF1* in leukemia [82] has also been reported; It has been demonstrated that a *GAS7*-mediated pathway suppressed proliferation of HCC cells following treatment with oxaliplatin, an alkylating anti-neoplastic agent, and the inhibition of *GAS7* negated the beneficial effects of the drug [83]. Similarly, NRG1 is associated with promoting metastasis of HCC cells by increasing epithelial-mesenchymal transition, thus increasing migratory behavior of the cancer cells [84] (genetic variants in *NRG1* have also been associated with schizophrenia [85]).

We observed some unexpected HBV integrations only in normal tissues but not in paired tumor tissues. Most of these integrations had very low integration allele fractions (<5%), including those located in cancer-related genes. For example, we found an unexpected integration in the *MAGI1* gene only in a normal tissue; however, only two chimeric and split reads were observed for this integration and its integration allele fraction was only 1.04%. *MAGI1* has been associated with HCC [34]. We observed another unexpected integration in the intronic region of *RTN4* only in a normal tissue, though its integration allele fraction was only 2.35%. *RTN4* has been demonstrated to induce apoptosis in cancer cells but play an opposite role in normal cells [32]. Similarly, we found an unexpected integration in the intronic regions of *EWSR1* only in normal tissue, with an integration allele fraction of only 3.13%. *EWSR1* has been implicated in pancreatic cancer [67]. We also found an integration (integration allele fraction was 1.61%) only in normal tissue in *DACH2*, a biomarker for muscle-invasive urothelial carcinoma [68]. Further analyses are needed to verify these potential integration events, including, but not limited to, the use of single cell sequencing to study tissue microenvironment [86,87]. The observations of integrations in normal tissues in *MAGI1*, *RTN4*, *EWSR1*, and *DACH1* highlight the need to screen larger cohorts of healthy tissues in future studies, similar to the current biobank efforts, which may distinguish viral integrations involved in early stages of tumorigenesis from random viral integration events.

We also observed that the lengths of integrated HBV sequences in tumor tissues were generally shorter than those integrated in normal tissues (Figure 2D). Further studies are needed to better understand how HBV integrations play a role in HCC, including lengths of HBV integrations, functional roles of inserted HBV DNA regions, cellular proportions of HBV integrations, and other covariates or factors. For example, it is known that the HBV X region is highly correlated with HCC onset [88]. We found that many HBV integrations in tumor tissues contained HBV enhancer or promoter regions, including parts of the HBV X region [13]. Similar research has been shown in Merkle cell carcinoma. Before Merkel cell polyomavirus can transform cells, truncated large T antigen gene sequences integrate into the host genome, leading to cellular transformation and tumor proliferation [88]. In addition, the HBV integrations in tumors had higher integration allele fractions (Figure 3A,B). By comparison, although the lengths of HBV integrations in normal tissues were longer, the total number of integration events was smaller, and their integration allele fractions were also lower. These results together further support the importance of including our proposed integration allele fraction analysis in studies aimed at identifying oncoviruses and cancer-related genes.

Furthermore, our pathway analysis of the novel non-recurrent genic HBV integrations revealed many critical metabolic pathways potentially involved in HCC. Notably, many pathways involving *ADH4* were among the most enriched hits. Proper *ADH4* functions are critical for alcohol metabolism [89], and functional changes in *ADH4* [90,91] and other alcohol dehydrogenase-related genes [92,93] are associated with alcohol dependence or alcohol-induced medical diseases. Alcohol is classified as group 1 carcinogen [94]. Excessive intake of alcohol leads to fatty liver, cirrhosis, and eventual development of HCC [94,95,96]. Moreover, many recent publications have shown that aberrations in *FAK* expression contribute to the onset and invasiveness of HCC [97,98,99], which is supported by our findings of an enrichment of the focal adhesion pathway involving genes such as *ITGA11*, *VCL*, and *LAMA*.

The applications of the VIcaller method and platform are not limited to analysis of HBV integrations or HCC but can be directly applied to detect other oncoviruses in other cancer types. Approximately 15% of the global cancer incidences have viral etiology [100]. For example, EBV infections, detected in more than 95% of the world population, are associated with multiple cancers, such as non-Hodgkin’s lymphoma, nasopharyngeal carcinoma, and gastric cancer [101]; also, approximately 99% of cervical cancer cases are associated with human papillomaviruses, as are 50% of penile, vulvar, and vaginal cancers [88]. Discovering new oncogenic effects of human viruses is critical since such findings may eventually lead to development of vaccination strategies to reduce virus-mediated cancer mortality. VIcaller allows using cancer genome data for identifying virome-wide viral infections, particularly integration sites and their fusion transcripts, and for determining early-stage clonal integrations involved in early-stage tumorigenesis. For instance, we found that HBV integrations in the *TERT* promoter regions were prone to have the same orientation as *TERT* (e.g., 22 out of the 29 integration events were in the same 3′ to 5′ orientation as *TERT* in the human genome). This supports possible formation of fusion transcripts, although other mechanisms [102,103,104] may also be possible. This also emphasizes the importance of examining sequence orientations of viral integrations and identifying both upper and lower breakpoints when possible.

For future research, it will be worthwhile to further investigate the precise HBV genotypes (i.e., 10 HBV strains from A-J [105]), since the epidemiological and pathological HBV strain variations may impact the progression and clinical outcomes of HCC [106]. The HBV subtypes [107] can be identified by employing the available online genotyping tools such as HBVdb, HBV STAR or HepSEQ. Furthermore, a multidisciplinary analysis, such as integrated genomic, transcriptomic (e.g., long-read DNA and RNA sequencing), epigenomic, and proteomic investigations, may provide further functional validation of the biological roles of viral integrations in human diseases and health.

## Figures and Tables

**Table 1:** Characterization of all the novel non-recurrent genic HBV integrations identified by VIcaller.

Integration Breakpoint (hg19)	SampleID	Annotation	Gene	Protein Functions	Associated Diseases or Biological Functions
chr1:34,234,244	154T	Intronic	*CSMD2*	CUB and sushi domain	Colorectal cancer [23], Schizophrenia [29]
chr1:47,605,956	95T	Intronic	*CYP4A22*	Cytochrome P450	BMI [30]
chr1:200,315,868	126N	Intronic	*LINC00862*	Non-coding RNA	NA
chr1:228,859,567	272T	Intronic	*RHOU*	Ras homolog family member U	Basal cell carcinoma [31]
chr2:55,204,658	145N	Intronic	*RTN4*	Reticulon family protein	Tumor suppressor [32]
chr2:216,294,876	152T	Intronic	*FN1*	Soluble fibronectin-1 released by liver to bloodstream for injury repair	Kidney disease [33]
chr2:216,300,026	128N	Intronic	*FN1*	Soluble fibronectin-1 released by liver to bloodstream for injury repair	Kidney disease [33]
chr3:65,589,091	154N	Intronic	*MAGI1*	Membrane associated kinase	Liver cancer [34], Depression [35]
chr4:74,270,112	30N	Coding	*ALB*	Albumin gene	NA
chr4:94,309,472	154N	Intronic	*GRID2*	Glutamate receptor	Cerebellum [36]
chr4:100,062,273	13N	Intronic	*ADH4*	Alcohol dehydrogenase	Alcohol dependence [37]
chr4:146,711,425	272T	Intronic	*ZNF827*	Zinc finger protein 827	Liver enzyme levels [38]
chr5:1,016,647	126T	Intronic	*NKD2*	Wnt antagonist	Multiple cancers [25]
chr5:59,475,207	75T	Intronic	*PDE4D*	Phosphodiesterase	Breast cancer [39]
chr5:128,849,386	154N	Intronic	*ADAMTS19*	ADAM metallopeptidase	Ovarian failure [40]
chr7:34,428,498	39N	Intronic	*NPSR1-AS1*	ncRNA gene	Asthma [41]
chr7:117,841,554	67N	Coding	*LSM8*	U6 Small nuclear RNA	Arthritis [42]
chr8:22,552,347	62T	Upstream	*EGR3*	Cell growth	Gastric cancer [43], Heart disease [44]
chr8: 25,207,744	67N	Intronic	*DOCK5*	Dedicator of Cytokinesis 5	Glucose homeostasis [45]
chr8:42,258,852	75T	Intronic	*VDAC3*	Voltage dependent anion channel	Mitochondrial dysfunction [46]
chr8:53,536,417	26N	Intronic	*RB1CC1*	RB1-induced coiled coil	Obesity [47]
chr8:85,567,668	14N	Intronic	*RALYL*	RALY RNA protein binding-like	Arthritis [42]
chr8:99,484,881	75T	Intronic	*STK3*	Serine/threonine kinase	Cell death [48], Heart disease [49]
chr8:101,731,867	71N	Intronic	*PABPC1*	Poly(A) binding protein	Duodenal cancer [50]
chr8:105,493,714	75T	Downstream	*MIR548A3*	Micro-RNA gene	NA
chr8:109,067,155	75T	Intronic	*RSPO2*	Respondin 2	Pancreatic cancer [51]
chr9:28,182,065	181T	Intronic	*LINGO2*	leucine rich repeat and Ig domain containing	Parkinson’s disease [52]
chr9:74,744,593	180N	Intronic	*GDA*	Guanine deaminase	Suicidal ideation [53]
chr10:75,807,552	108N	Intronic	*VCL*	Vinculin	IBD [54]
chr10:96,798,621	126N	Intronic	*CYP2C8*	Cytochrome P450 family	Cancer drug metabolism [55], Nerupathy [55]
chr11:33,797,296	101N	Intronic	*FBXO3-AS1*	NA	NA
chr12:47,178,345	101N	Intronic	*SLC38A4*	Solute carrier family	Prostate cancer [56]
chr12:122,312,273	106T	Intronic	*HPD*	4-Hydroxyphenylpyruvate Dioxygenase	Blood metabolites [57]
chr13:53,024,234	29T	Coding	*VPS36*	Vacuolar Protein Sorting	Dementia [58]
chr14:73,450,075	145T	Intronic	*ZFYVE1*	Protein recruitment in membrane trafficking	Autophagasome [59]
chr14:76,267,746	70N	Intronic	*TTLL5*	Tubulin tyrosine ligase like 5	Retinal dystrophy [60]
chr15:68,603,361	70N	Intronic	*ITGA11*	Integrin subunit	Lung cancer [61]
chr16:59,570,392	26N	Upstream	*RNU4-58P*	Pseudogene	NA
chr16:65,533,699	261N	Intronic	*LINC00922*	NA	NA
chr17:30,697,197	39N	Coding	*ZNF207*	Zinc finger protein	NA
chr17:66,267,160	154T	Coding	*SLC16A6*	Monocarboxylic acid transmembrane transport; Lysosomal protein	Encephalopathies [62], Blood pressure [63]
chr17:66,267,160	154T	Intronic	*ARSG*	NA	NA
chr18:6,968,987	117N	Intronic	*LAMA1*	Laminin subunit (extracellular matrix)	Cerebellar dysplasia [64]
chr18:6,968,987	117N	Downstream	*RN7SL537P*	Pseudogene	NA
chr19:23,429,885	75T	Intronic	*ZNF724P*	Pseudogene	NA
chr19:36,335,975	22N	Intronic	*NPHS1*	Nephrin	Kidney disease [65]
chr21:22,477,453	204N	Intronic	*NCAM2*	Neuronal cell adhesion molecule 2	Visceral fat [66]
chr22:29,683,267	71N	Intronic	*EWSR1*	RNA binding protein	Pancreatic cancer [67]
chrX:86,077,055	114N	Intronic	*DACH2*	Transcription factor	Bladder cancer [68]

NA represents no known functions or disease associations.

**Table 2:** Pathway enrichment analysis of all genes with non-recurrent HBV integrations.

Pathways	Genes	Enrichment Ratios	*p* Values (Adjusted)
Retinol metabolism	*ADH4*, *CYP2C8*, *CYP4A22*	43.95	0.0005
Tyrosine metabolism	*ADH4*, *HPD*	45.73	0.004
Fatty acid metabolism	*ADH4*, *CYP4A22*	43.61	0.004
Focal adhesion	*ITGA11*, *VCL*, *LAMA1*	14.06	0.004
Arachidonic acid metabolism	*CYP2C8*, *CYP4A22*	31.78	0.004
Drug metabolism-cytochrome P450	*ADH4*, *CYP2C8*	25.69	0.005
Metabolism of xenobiotics by cytochrome P450	*ADH4, CYP2C8*	26.41	0.005
ECM-receptor interaction	*ITGA11*, *LAMA1*	22.06	0.006
Amoebiasis	*VCL*, *LAMA1*	17.69	0.008
Purine metabolism	*GDA*, *PDE4D*	11.57	0.01
Regulation of actin cytoskeleton	*ITGA11*, *VCL*	8.8	0.02

The *p* values were adjusted for false discovery rate using the Benjamini and Hochberg method.

## Supplementary Materials

The following are available online at https://www.mdpi.com/1999-4915/13/2/245/s1, Figure S1: HBV integrations detected in repeat regions, Figure S2: comparison of numbers of supporting reads, Figure S3: comparison of upper and lower breakpoints, Figure S4: comparison of numbers of HBV integrations.
