# Supplementary material for: Characterization of Hepatitis B Virus Integrations Identified in Hepatocellular Carcinoma Genomes
Source: Viruses. 2021 Feb 4;13(2):245. doi: 10.3390/v13020245 (PMC7915589; doi:10.3390/v13020245)
Supplement: Supplementary file 1 [file viruses-13-00245-s001.zip › viruses-1080968 Supplementary Figure Legends.pdf]

Supplements

Supplementary Figure Legends

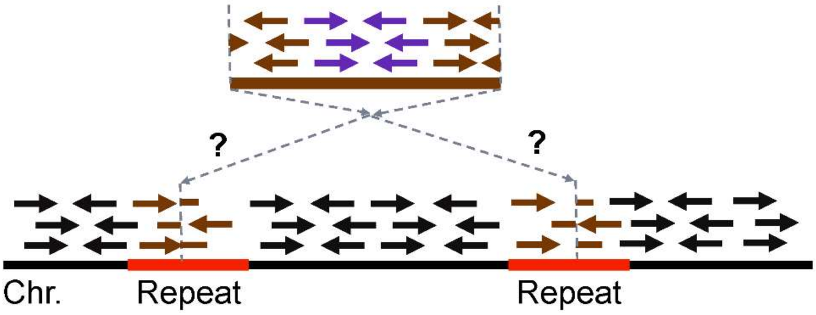

Supplementary Figure 1 HBV integrations detected in repeat regions tend to be less accurate because of multiple mapping. Bars in brown represent potentially inserted HBV sequences. Brown arrows represent the chimeric and split reads supporting HBV integrations in repeat regions. Purple arrows represent HBV reads. Dark grey arrows represent the reads mapped to the human genome supporting no integrations.

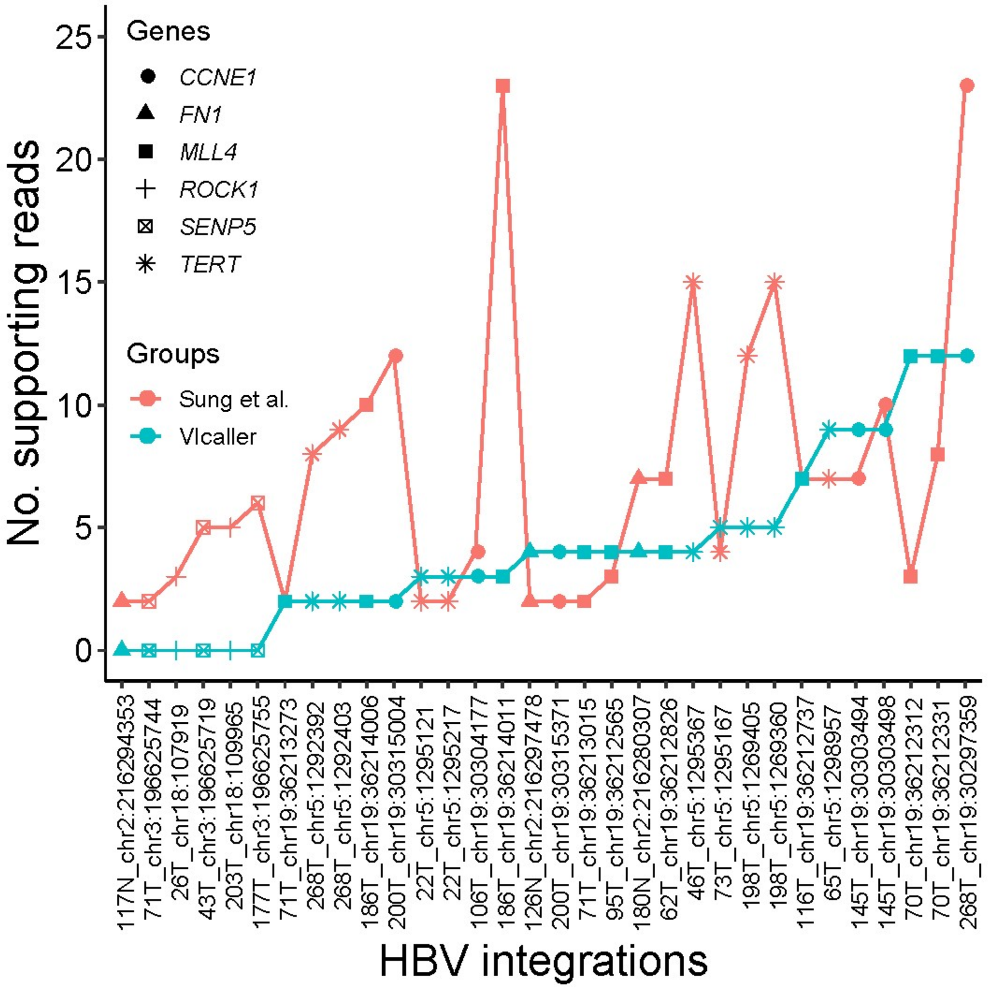

Supplementary Figure 2 Comparison of the number of supporting reads (including chimeric and split reads) of the 32 PCR-validated HBV integrations detected by both VIcaller and Sung *et al*. Among these 32 integrations, VIcaller detected more chimeric and split reads for 11 integrations, the same number of reads for two integrations, and fewer reads for 19 integrations than those identified in the Sung *et al*. study.

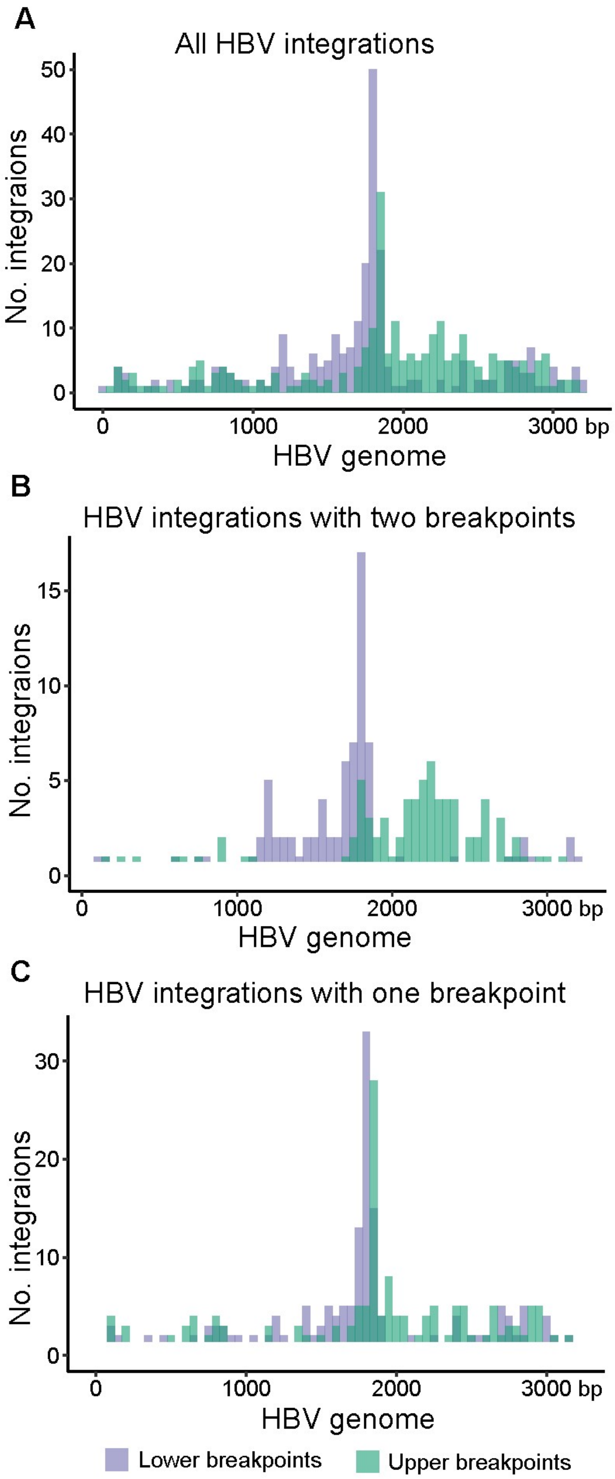

Supplementary Figure 3 Comparison of upper and lower breakpoints on the HBV genome for **A**) all HBV integrations, **B**) HBV integrations with two breakpoints, and **C**) HBV integrations with one breakpoint.

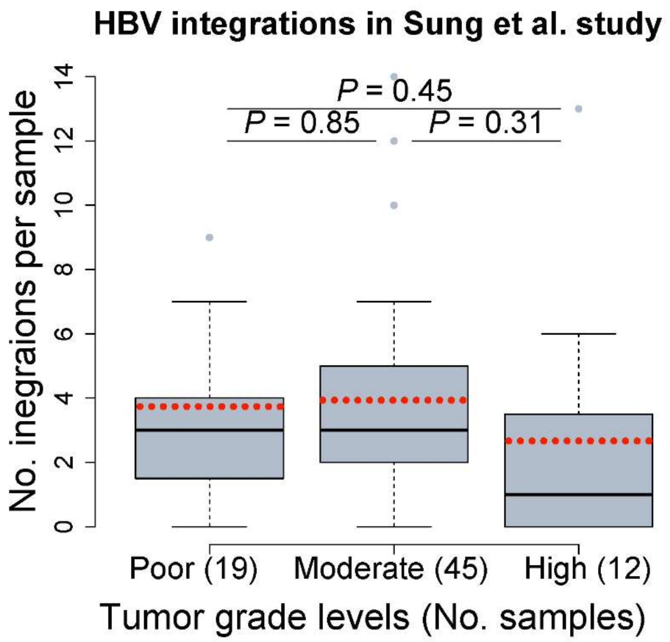

Supplementary Figure 4 Comparison of numbers of HBV integrations detected by Sung *et al* among poor, moderately, and highly differentiated tumors. Student's *t* test was used to compare each group pair. The dotted red line indicates the average number of HBV integrations for each patient group.
