# Supplementary figures and images for: Characterization of Hepatitis B Virus Integrations Identified in Hepatocellular Carcinoma Genomes
Source: Viruses. 2021 Feb 4;13(2):245. doi: 10.3390/v13020245 (PMC7915589; doi:10.3390/v13020245)

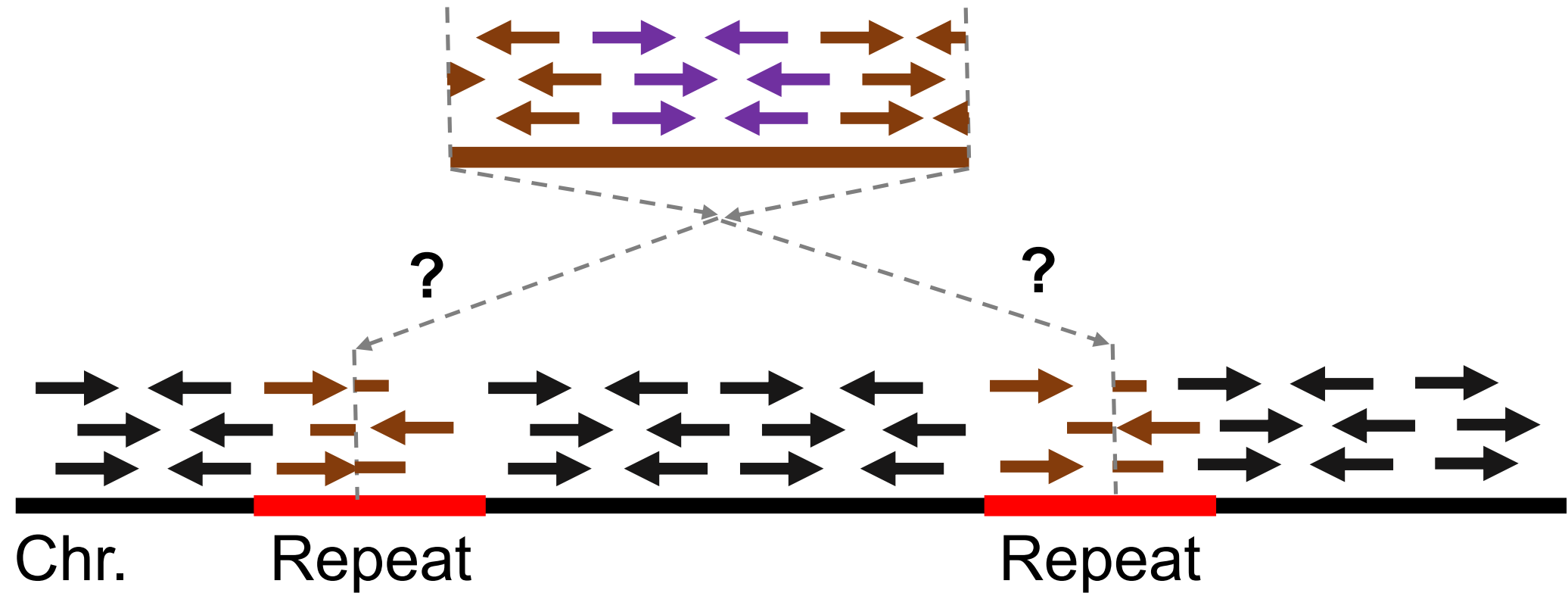

Supplement: Supplementary file 1 [file viruses-13-00245-s001.zip › Supplementary Figure 1.pdf]

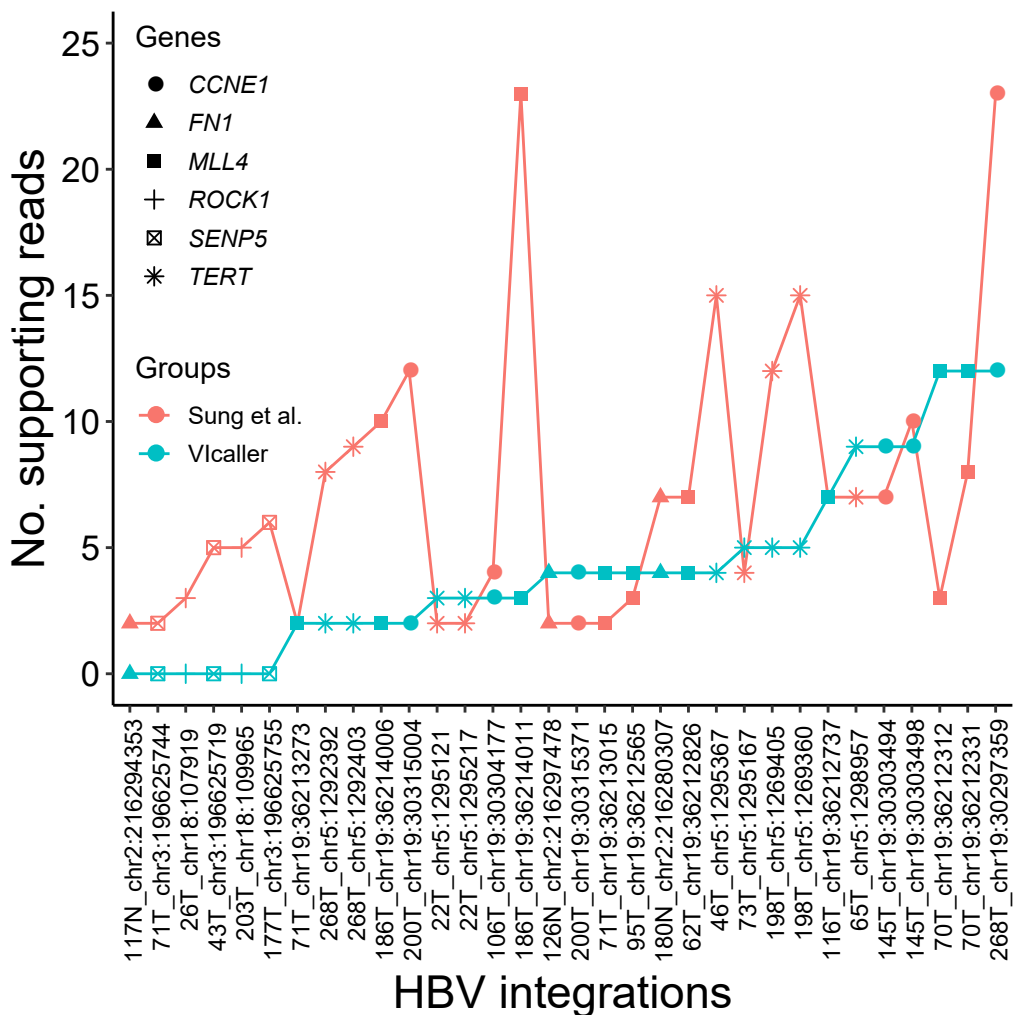

Supplement: Supplementary file 1 [file viruses-13-00245-s001.zip › Supplementary Figure 2.pdf]

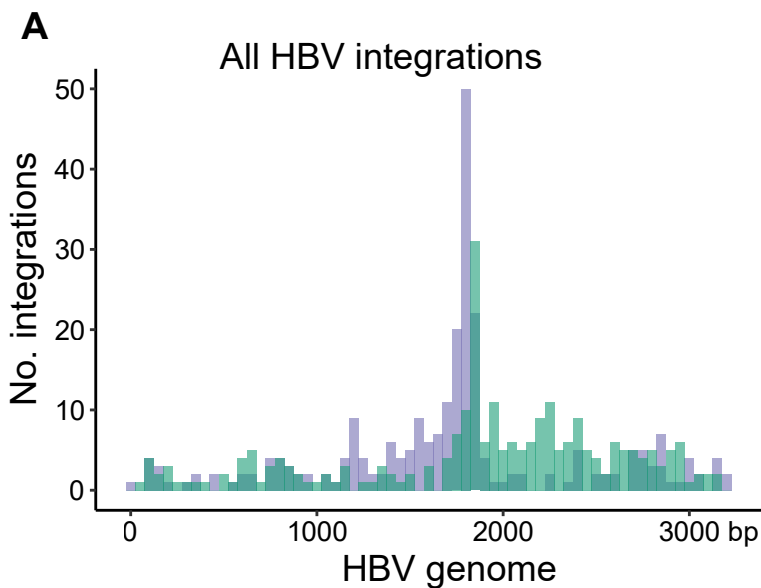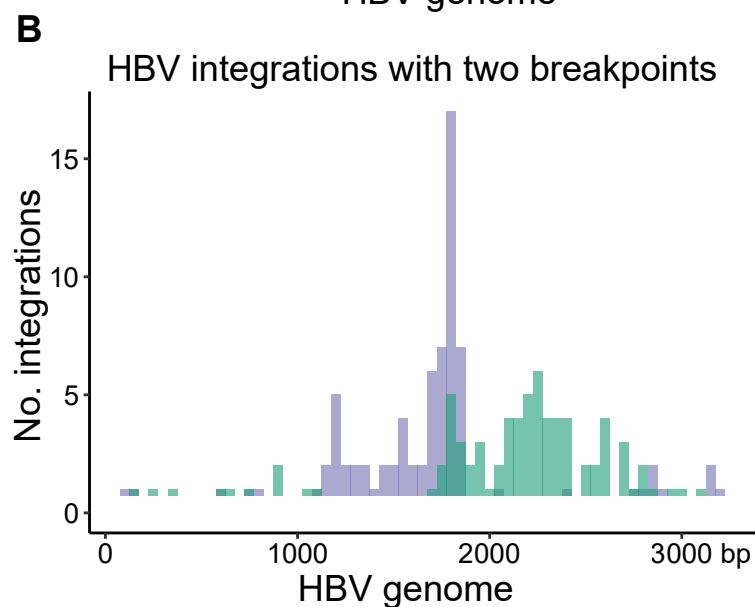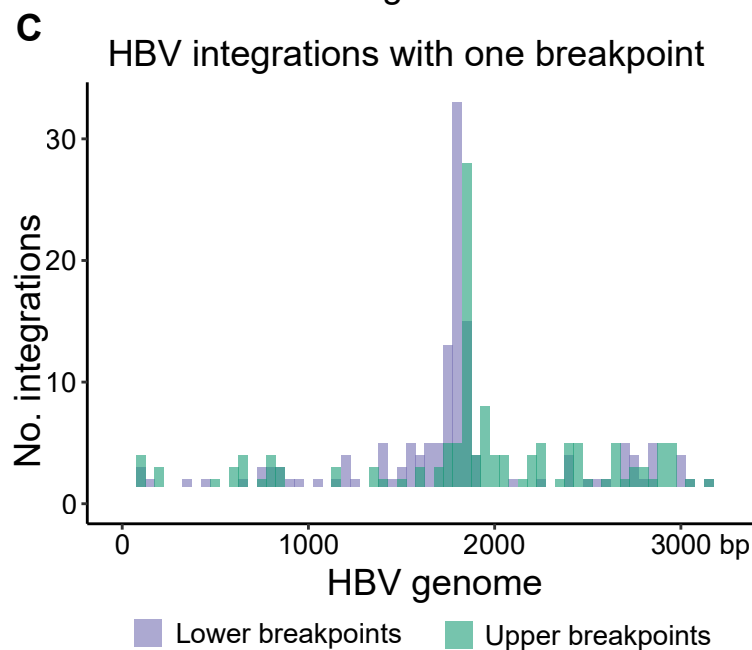

Supplement: Supplementary file 1 [file viruses-13-00245-s001.zip › Supplementary Figure 3.pdf]

# HBV integrations in Sung et al. study

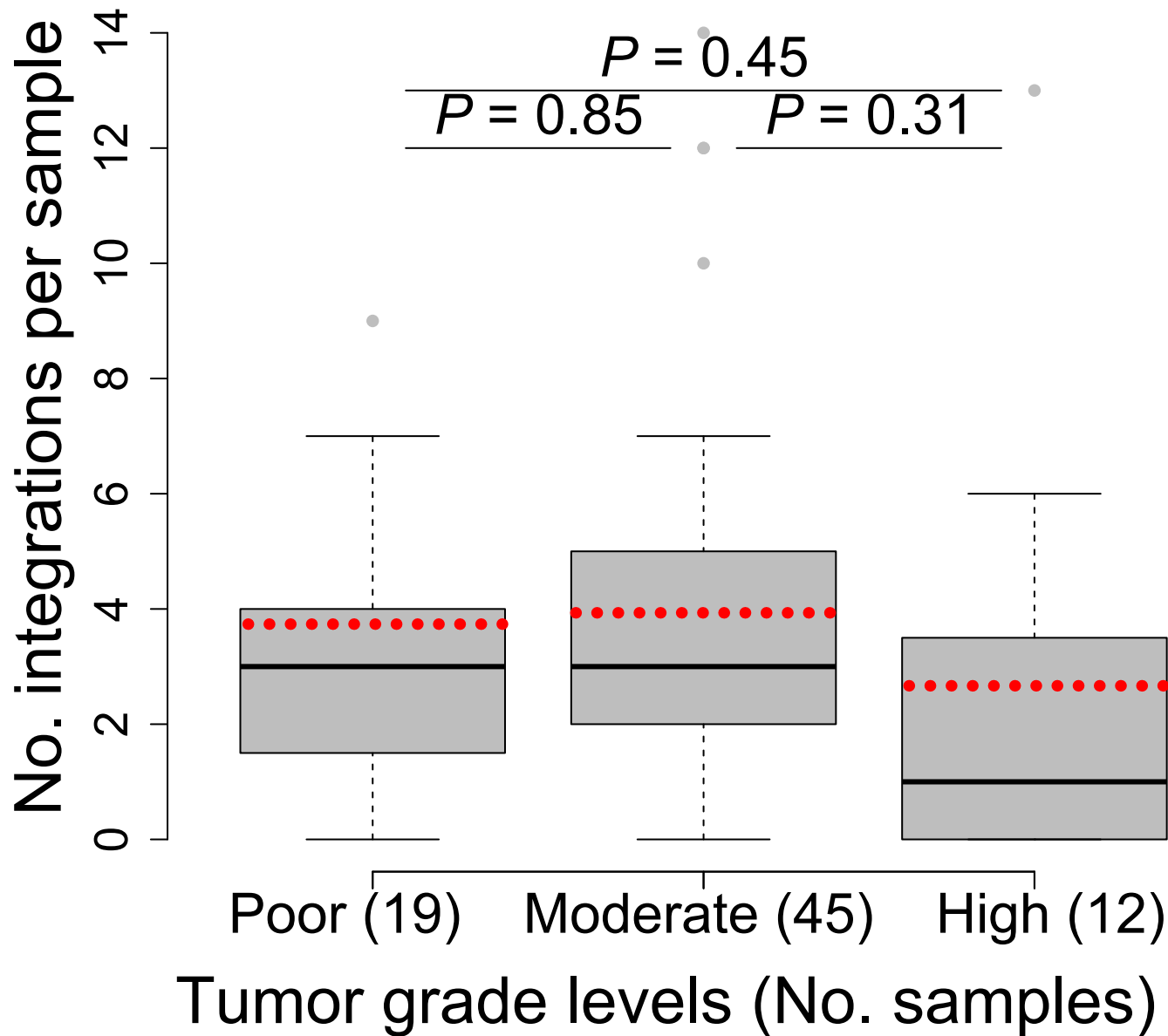

Supplement: Supplementary file 1 [file viruses-13-00245-s001.zip › Supplementary Figure 4.pdf]
